# Preparation of a Novel Millet Straw Biochar-Bentonite Composite and Its Adsorption Property of Hg^2+^ in Aqueous Solution

**DOI:** 10.3390/ma14051117

**Published:** 2021-02-27

**Authors:** Yanzhen Bai, Jianping Hong

**Affiliations:** College of Resources and Environment, Shanxi Agricultural University, Taigu District, Jinzhong 030801, China; yanzhenbai@163.com

**Keywords:** biochar, bentonite, composite, Hg^2+^, aqueous solution

## Abstract

The remediation of mercury (Hg) contaminated soil and water requires the continuous development of efficient pollutant removal technologies. To solve this problem, a biochar–bentonite composite (CB) was prepared from local millet straw and bentonite using the solution intercalation-composite heating method, and its physical and chemical properties and micromorphology were then studied. The prepared CB and MB (modified biochar) had a maximum adsorption capacity for Hg^2+^ of 11.722 and 9.152 mg·g^−1^, respectively, far exceeding the corresponding adsorption value of biochar and bentonite (6.541 and 2.013 mg·g^−1^, respectively).The adsorption of Hg^2+^ on the CB was characterized using a kinetic model and an isothermal adsorption line, which revealed that the pseudo-second-order kinetic model and Langmuir isothermal model well represented the adsorption of Hg^2+^ on the CB, indicating that the adsorption was mainly chemical adsorption of the monolayer. Thermodynamic experiments confirmed that the adsorption process of Hg^2+^ by the CB was spontaneous and endothermic. Fourier transform infrared spectroscopy (FTIR), X-ray photoelectron spectroscopy (XPS), and a thermogravimetric analysis (TGA) showed that after Hg^2+^ was adsorbed by CB, functional groups, such as the –OH group (or C=O, COO–, C=C) on the CB, induced complexation between Hg and –O–, and part of Hg (ii) was reduced Hg (i), resulting in the formation of single or double tooth complexes of Hg–O– (or Hg–O–Hg). Therefore, the prepared composite (CB) showed potential application as an excellent adsorbent for removing heavy metal Hg^2+^ from polluted water compared with using any one material alone.

## 1. Introduction

Mining smelting and sewage irrigation can enhance the accumulation of heavy metal mercury in farmlands and water bodies. Mercury can migrate to crops through sewage irrigation, which can have adverse effects on human health [1]. To solve this problem, the environmental mercury content is controlled by the use of such methods as precipitation [2], adsorption [3], ion exchange [4], bioremediation [5], electric remediation [6], and flocculation [7]. Among these methods, an increasing amount of attention is being paid to the use of adsorbents because of their effectiveness and ease of operation. For example, Tan demonstrated an effective method for removing heavy metal mercury from wastewater by using carbon-based adsorbents [8]. Yadav demonstrated an effective method for removing heavy metal mercury from wastewater by using clay minerals such as bentonite [9].

Biochar is a type of substance produced by the slow pyrolysis of organic substances at high temperature, which can be used as an adsorbent to fix heavy metals and reduce toxicity. However, the ability of biochar to remove pollutants from aqueous solution is limited, and further modification is needed to enhance its adsorption performance. Recently, the synthesis of biochar-based metal oxide complexes for removing heavy metals from aqueous solutions has been studied by other scholars [10,11]. Although this type of composite has higher adsorption performance than separate biochar, there are two disadvantages: one is that the metal element is expensive and the cost of the synthesized material is high, and the other is that there is another metal in the composite, which may cause secondary pollution due to the release of metal in the adsorbent during the process of adsorbing pollutants. Therefore, these two shortcomings make it difficult to popularize the composite. Therefore, a composite of non-metallic materials and biochar will have more advantages than a composite of metallic materials and biochar for adsorbing pollutants. However, there exist few studies regarding this combination.

Previous studies [8,9] have shown that biochar and bentonite, as adsorbents, have the ability to adsorb heavy metals, but there are few reports on whether they can improve the adsorption effect after synthesis. Currently, most of the studies regarding heavy metals in the environment have focused on the adsorption of heavy metals such as Cd^2+^ and Cu^2+^ [12], and studies on Hg^2+^, which is more toxic, are relatively fewer. There are only a few studies on the adsorption of ions by composite materials; only the adsorption of copper [13], zinc [14,15], chromium [16], and lead [17] ions in water has been studied. However, the adsorption of the more toxic Hg^2+^ has not been reported so far. Based on the above considerations, it is particularly necessary to study the adsorption performance of a biochar–bentonite composite for Hg^2+^ and analyze its adsorption mechanism. In addition, the contribution of biochar and bentonite parts in composite materials to the removal of mercury ions from water should also be discussed together.

Therefore, this study was devoted to the preparation of a biochar–bentonite natural non-toxic composite and its effect and mechanism of mercury ion removal from aqueous solution. The specific objectives are as follows: (1) to study the adsorption characteristics of the CB for Hg^2+^ in an aqueous solution; and (2) to characterize the CB before and after Hg^2+^ adsorption using FTIR, XPS, and TGA so as to clarify the mechanism of Hg^2+^ removal.

## 2. Materials and Methods

### 2.1. Materials

All of the chemicals were analytical grade reagents. The hydrochloric acid was purchased from the Beijing Chemical Plant, China, and the potassium hydroxide was purchased from the Tianjin Kemeo Chemical Reagent Co., Ltd. (Tianjin, China). A standard solution of mercury (100 mg/L) was obtained from the Institute of Reference Materials of the Ministry of Ecology and Environment of China (National Reference Materials Center). Bentonite was purchased from the Huachen Mineral Products Trading Co., Ltd., (Shijiazhuang, China). Millet straw was collected from Sunzhuang Village, Shahe Town, Fanshi County, Xinzhou City, Shanxi Province, China, and the variety was Jingu 22. The straw was harvested after 120 days of growth, dried at 65 °C, and crushed.

### 2.2. Preparation of the Adsorbent and Process Optimization

#### 2.2.1. Preparation of the CB

There are four kinds of adsorbents, namely biochar (B), modified biochar (MB), bentonite (BE), and biochar–bentonite composite (CB). The specific preparation process is as follows:

Dry millet straw was ground through a 200 mesh sieve (MS) and slowly pyrolyzed in a muffle furnace at 300 °C for 2 h under the protection of N_2_ (0.5 L·min^−1^) to prepare the biochar (B).

Then 3 mol·L^−1^ of a KOH solution (millet straw/g: KOH/mL = 1: 30) was added to 8 g of the MS mixture and shaken for 30 m to form a uniform suspension. The mixture was centrifuged (4248× *g*, 5 min) after the reaction was conducted for 2 h using constant stirring. The product was dried at 105 °C, ground, and sieved. The product was soaked in 3 mol·L^−1^ of hydrochloric acid and boiled for 1 min. After cooling at room temperature, the product was washed and boiled to neutrality using deionized water. Then the product was dried to a constant weight at 110 °C, ground, and passed through a 200 mesh sieve. Under the protection of N_2_, the modified biochar (MB) was prepared using slow pyrolysis at 300 °C for 2 h in a muffle furnace.

A total of 3 mol·L^−1^ of a KOH solution (millet straw/g: KOH/mL = 1: 30) was added to 8 g of the MS mixture and shaken for 30 min to form a uniform suspension. Then, 4.0 g of bentonite (BE) was added into 120 mL distilled water and shaken for approximately 30 min, after which it was slowly added into the KOH suspension containing the millet straw powder; the temperature was raised to 70 °C, and it was stirred continuously and allowed to react for 24 h. The mixture was then centrifuged (4248× *g*, 5 min), and the product was dried at 105 °C. The product was soaked and boiled with 3 mol·L^−1^ of hydrochloric acid for 1 min. After cooling at room temperature, the product was washed and boiled to neutrality using deionized water. Then the product was dried at 110 °C to a constant weight. Finally, it was ground and passed through a 200 mesh sieve. Under the protection of N_2_, the millet straw biochar composites (CB) were prepared using slow pyrolysis at 300 °C for 2 h in a muffle furnace.

#### 2.2.2. Process Optimization

To determine the influence of synthesis conditions on the CB adsorption, an orthogonal test L_27_ (3^13^) was designed based on the adsorption capacity of Hg^2+^. The particle size of the straw, the mass concentration of the KOH solution, the mixing ratio of the straw biomass and bentonite, the reaction temperature of the biomass and bentonite, and the carbonization temperature of the CB were then investigated. See the supplementary materials (Tables S1 and S2; Figure S1) for details.

### 2.3. Characteristics of the Adsorbent

The total contents of carbon, hydrogen, oxygen, nitrogen, and sulfur in the absorbent were determined using an elemental analyzer (Vario Macrocube Elementar, Langenselbold, Germany). The ash content was measured by heating the sample at 550 °C for 2 h. An X-ray photoelectron spectrometer (ESCALAB 250Xi, Thermo Fisher Scientific, Waltham, MA, USA) was used to determine the surface chemical composition. The mercury content in the CB was determined using a direct mercury analyzer (DMA-80, MileStone, Bergamo, Italy). A scanning electron microscope (JSM-6490LV, JEOL, Tokyo, Japan) was used to examine the surface physical morphology of the samples. The functional groups of the CB were characterized using a Fourier transform infrared spectrometer (Tensor27, Bruker, Germany). The BET (Brunauer, Emmett, and Teller) surface area, total pore volume, and pore size distribution of the adsorbent were measured using the nitrogen adsorption at 77 K using a specific surface area meter (ASAP2020, Micromeritics, Norcross, GA, USA). The thermal stability of the CB was measured using a synchronous thermal analyzer (Lab Sys evo, Setaram, Lyon, France).

### 2.4. Adsorption Experiments

The adsorption experiment was conducted in a 100 mL triangular flask at 25 °C. The initial concentration of Hg^2+^ in the solution was approximately 1 mg·L^−1^. A total of 20 mg of the adsorbent and 20 mL of the Hg^2+^ solution were mixed in a triangular flask. The initial pH of the solution was adjusted to 7.0 using a 0.1 mol·L^−1^ of sodium hydroxide and hydrochloric acid solution. Then 0.01 mol·L^−1^ CaCl_2_ was added to the mercury solution to control ionic strength. The triangular flask was sealed and shaken for 60 min at a speed of 180 rpm in a rotary shaking incubator to achieve apparent equilibrium. Then, the solution was diluted and centrifuged, and the concentration of Hg^2+^ in the supernatant was determined using a direct mercury analyzer.

In the isothermal adsorption experiment, the initial concentration of Hg^2+^ in the solution was in the range of 0.5–20 mg·L^−1^, and the rest was the same as above. In the kinetic adsorption experiment, the oscillation time was controlled within 1–60 min, and the rest were the same as above. Thermodynamics, the experimental temperature was set at 15, 25, and 35 °C, and the experimental process was the same as the isothermal adsorption experiment. The effect of initial pH was studied by adjusting the pH of the solution to 2–8 with 0.1 mol·L^−1^ sodium hydroxide/hydrochloric acid solution.

All of the adsorption experiments were performed in triplicate. The repeated experiment, blank experiment, and control experiment were used to control the experimental error. In the control experiment, Chinese national reference materials (water standard sample, 202035 and geological soil sample, GSS-23) were used for experimental control.

### 2.5. Data Analysis

The adsorption capacity is expressed by the following equation.

q = (C_0_ − Ce)V/m (1)

In the formula, q is the equilibrium capacity (mg·g^−1^), C_0_ and C_e_ are initial concentration and equilibrium concentration of Hg^2+^ (mg·L^−1^), respectively, V is the volume of metal solution (L), and m is the dosage of adsorbent (g).

The pseudo-first-order and second-order dynamic model equations are expressed using the following equations, respectively:

dQt/dt = k1·(qe − Qt)(2)

dQt/dt = k2·(qe − Qt)^2^ (3)

where Qt is the adsorption capacity (mg·g^−1^) at t (min); qe is the theoretical equilibrium adsorption capacity (mg·g^−1^); and k1 and k2 are the pseudo first- and second-order adsorption rate constants (min^−1^), respectively.

Both the Freundlich model and the Langmuir model were used to describe adsorption isotherms. These two models are represented by the following equations:

Qe = b·Q_max_·Ce/(1 + b × Ce) (4)

Qe = Kf·Ce^1/n^ (5)

where Qe and Ce are the equilibrium adsorption capacity (mg·g^−1^) and equilibrium concentration (mg·L^−1^), respectively; Qmax is the theoretical maximum adsorption capacity; and b is Langmuir constant and Kf is Freundlich constant, which can be used to characterize the adsorption capacity of adsorbent; 1/n is an empirical constant related to the adsorption capacity and the adsorption density.

The adsorption thermodynamics, that is, the energy change during the adsorption process, is primarily determined by calculating the thermodynamic parameters (the adsorption free energy change ΔG^0^, the adsorption enthalpy change ΔH^0^, and the adsorption entropy change ΔS^0^):

ΔG^0^ = − R·T·Ln(k) (6)

Ln (k) = − ΔH^0^/(R·T) + ΔS^0^/R (7)

where ΔG^0^ is the standard adsorption free energy change; ΔH^0^ is the standard adsorption enthalpy change; ΔS^0^ is the standard adsorption entropy change; k is the adsorption equilibrium constant; T is the absolute temperature of the system; and R is the ideal gas constant.

The analysis quality assurance and quality control of Hg were achieved using repeated experiments, a blank experiment, a reference substance experiment, a water standard sample (202035), and a geological soil sample (GSS-23). The standard sample was obtained from the standard sample of the National Reference Material Center. All measurements were repeated three times, and the average value was used in the data analysis.

The experimental data were statistically analyzed using Excel2017 and IBM SPSS22.0 and plotted using Origin 8.2. The combinations of all the XPS spectra were analyzed using XPSPEAK 41 software (XPSPEAK 41, Thermo Fisher Scientific, Waltham, MA, USA).

## 3. Results and Discussion

### 3.1. Characterization of the Adsorbent

The elemental and ash analysis results of the B, BE, MB, and CB are shown in Table 1, indicating that there were significant differences among these materials. The carbon content of the CB was 17.2%, which was significantly lower than that of the B (84.9%) and the MB (55.1%). The oxygen content of the CB was 28.5%, which was significantly higher than that of the B (17.6%) and the MB (21.2%), but less than that of the BE. The relative element content of hydrogen in the CB was less than that of the B and the MB. These data showed that the polarity and oxygen containing groups of the CB were greatly increased compared with the original biochar. The ash content of the CB was greater than the MB and the B. The above contents indicated that the carbon, hydrogen, and oxygen that originally belonged to the biochar and bentonite co-existed in the CB.

**Figure 1:**
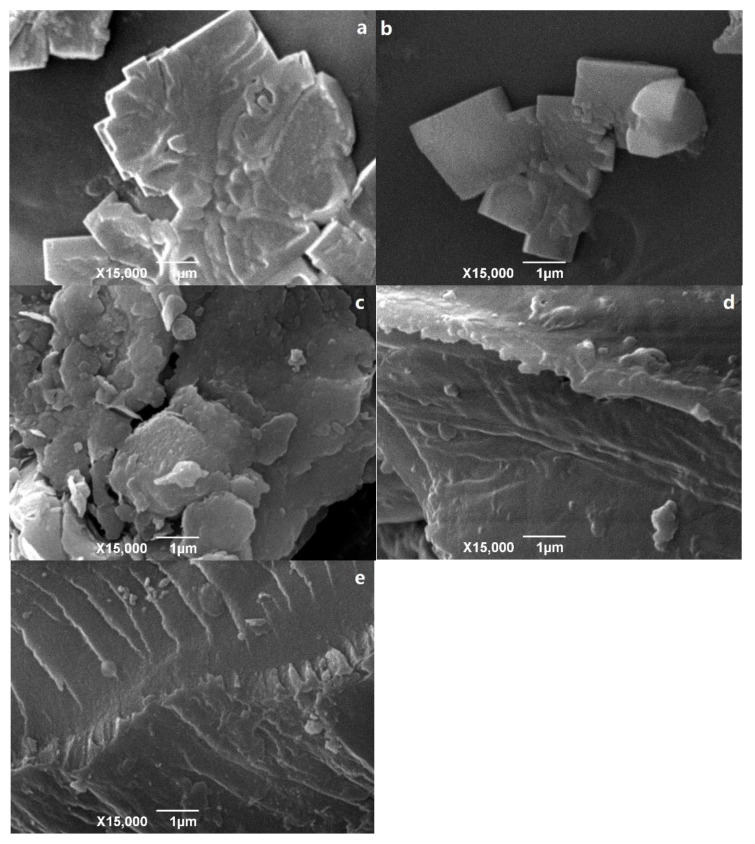
The SEM images of the B (**a**), MB (**b**), BE (**c**), CB (**d**), and the CB after Hg^2+^ adsorption (**e**).

The specific surface area, average pore diameter, and pore volume of the four materials measured using nitrogen adsorption (Table 1) revealed that the CB showed a mesoporous structure, while the B, BE, and MB showed macroporous structures. The respective specific surface areas of the CB and the MB (945.33 and 869.2 m^2^·g^−1^) were much larger than those of the B (86.7 m^2^·g^−1^). A similar increase in the surface area of the modified biochar has been observed in related studies [18]. Similar results were obtained by etching activated sludge biochar with potassium hydroxide [19]. The average pore width of the CB was less than the MB, which indicated that the composite product of the bentonite and biochar produced more micropores and also increased the surface area and pore volume of the CB. After the composite reaction of the polar biochar and nonpolar bentonite, some micropores may have been produced and changed from mesopores/macropores to micropores. The SEM images clearly show that the CB was composed of irregular plates and had a curled surface structure, thus providing effective adsorption sites (Figure 1).

**Figure 2:**
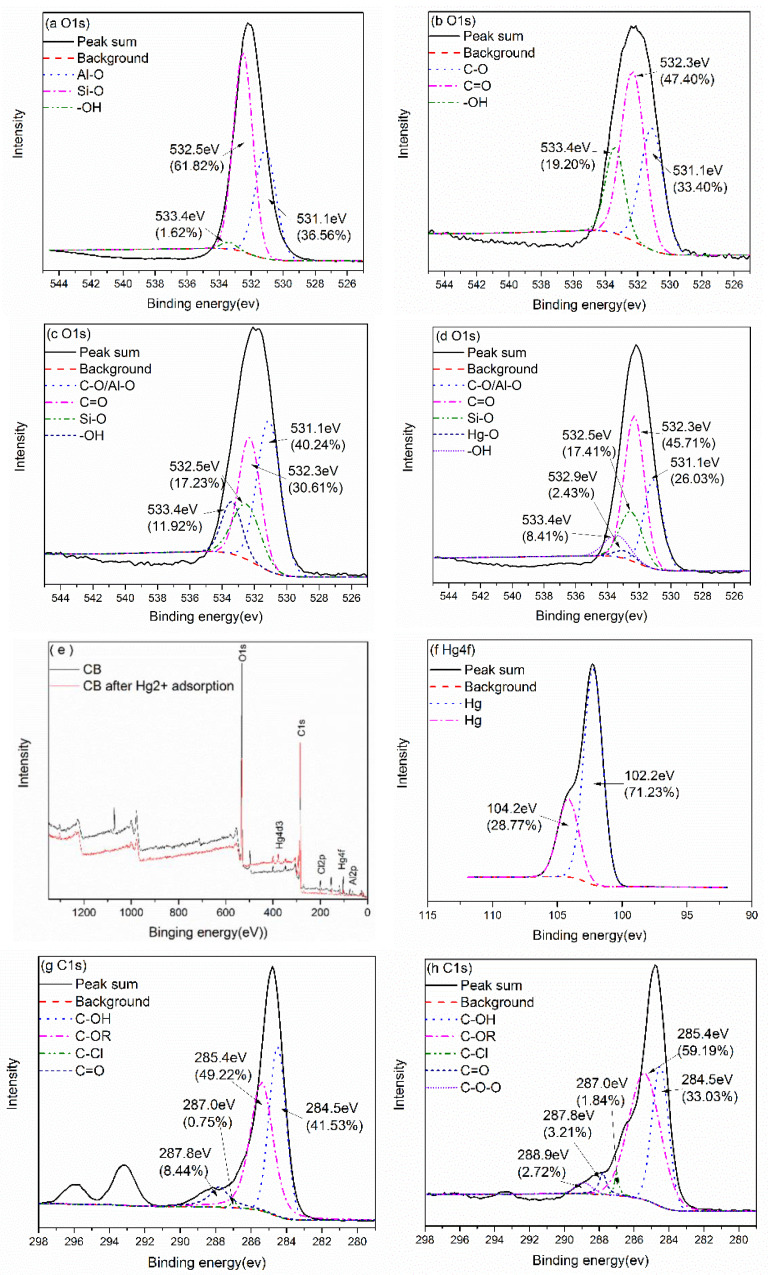
The XPS showing O1s core level in BE (**a**); XPS showing O1s core level in MB (**b**); XPS showing O1s core level in CB (**c**); XPS showing O1s core level in CB after Hg^2+^adsorption (**d**); XPS full survey of CB before and after Hg^2+^adsorption (**e**); XPS showing Hg4f core level in CB after Hg^2+^ adsorption (**f**); XPS showing C1s core level in CB (**g**); XPS showing C1s core level in CB after Hg^2+^ adsorption (**h**).

The state of oxygen incorporated in the CB samples was examined using XPS, and the spectra were compiled and are shown in Figure 2. The tail removed spectrum of the oxygen peak is shown in Figure 2. The binding energy of O (1s) was between 531.1 and 533.4 eV. After composite modification, the O (1s) content percentage of the CB was between that of the MB and the BE (Table 1). Figure 2a shows that most of the surface oxygen in the BE existed in the form of Al–O (35.56%) and Si–O (61.82%), which is a typical silicon–oxygen tetrahedron and aluminum–oxygen octahedron structure in bentonite [20]. Most of the surface oxygen in the MB (Figure 2b) existed in the form of C–O (33.40%) and C=O (47.40%). After composite modification, the O (1s) spectrum of the CB was divided into four peaks (Figure 2c) at 531.1, 532.3, 532.5, and 533.4 eV [21,22,23]. These peaks could be attributed to C–O/Al–O, C=O, Si–O, and –OH in the CB. Most of the surface oxygen was bound to the CB in the form of C–O/Al–O (40.24%) and C=O (30.61%), which provided abundant active adsorption sites for Hg^2+^. There was a small amount of oxygen in the MB in the form of –OH (19.20%). After composite modification, the molar ratio of this portion of oxygen was reduced to 11.92%. This portion of the active oxygen on the CB may have a strong binding ability for Hg^2+^.

### 3.2. Adsorption Performance of the CB on Hg^2+^

#### 3.2.1. Kinetic Adsorption of Hg^2+^ by the CB

**Figure 3:**
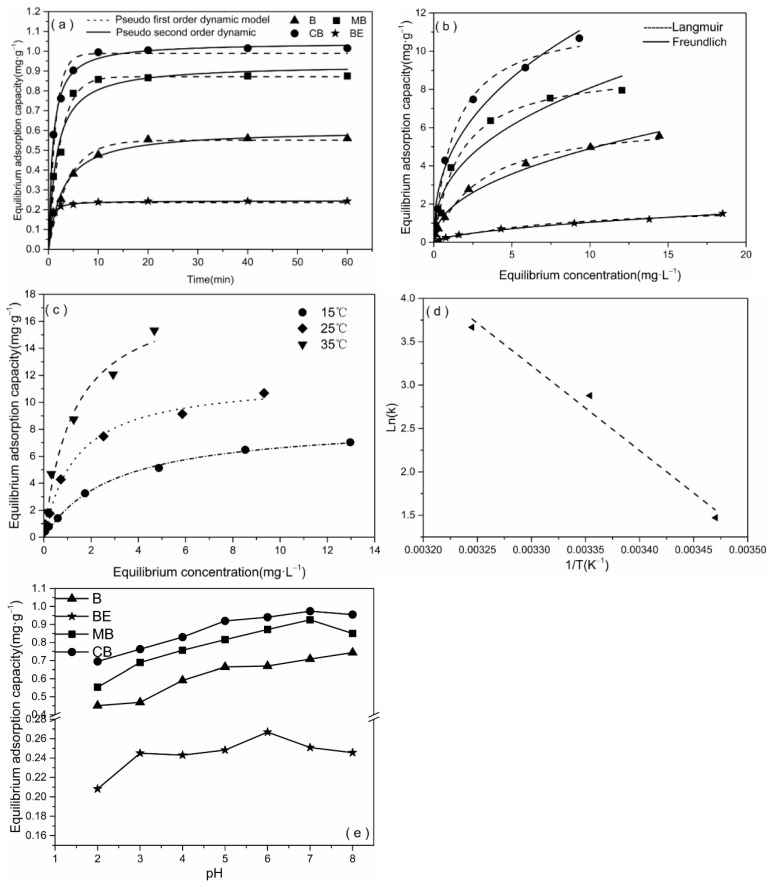
The sorption kinetics (**a**) and isothermal adsorption (**b**) of mercury onto the adsorbent. The thermodynamic adsorption of mercury by CB (**c**). The adsorption thermodynamics model of Hg^2+^ on CB was a linear regression (**d**). Effect of initial pH on the adsorption of Hg^2+^ onto the adsorbent (**e**).

The adsorption kinetics of mercury on the different adsorbents are shown in Figure 3a. The adsorption of the B, MB, and CB showed a rapid initial rate (<10 min) and then slowed down after 20 min until equilibrium. Under the equilibrium state, the qe value of mercury absorption showed the trend of CB > MB > B > BE. Table 2 summarizes the corresponding fitting parameters and determination coefficient values. For the B, BE, and CB, the R^2^ values of the pseudo-second-order kinetic model were 0.981, 0.994, and 0.936, respectively (Table 2), which were higher than those of the pseudo-first-order kinetic model (0.964, 0.959, and 0.916), so they had better fitting. This indicates that the adsorption of mercury by the B, BE, and CB was primarily a chemical adsorption process. The MB showed that the R^2^ (0.959) of the pseudo-first-order kinetic model was larger than that of pseudo-second-order kinetic model (0.936), which indicated that the etching of the biochar by the hydrochloric acid and potassium hydroxide only increased the number of micropores and specific surface area, and the adsorption of Hg^2+^ primarily enhanced the physical adsorption.

#### 3.2.2. Isothermal Adsorption of Hg^2+^ by the CB

Figure 3b shows the adsorption isotherms of Hg^2+^ on the B, BE, MB, and CB. The adsorption data were fitted using the Freundlich and Langmuir models, and the fitting parameters are listed in Table 3. The Langmuir model provided a better fit than the Freundlich model (R = 0.984—0.995 vs. R = 0.949—0.995, Table 3), which indicated that physical and chemical adsorption played a leading role in the adsorption of Hg^2+^. In previous studies, the Langmuir model has been typically used to fit the adsorption of heavy metal ions on the adsorbents, biomass, and graphene carbon [24]. Therefore, the adsorption data are primarily discussed here based on the fitting results of the Langmuir model.

Among the four materials, the CB had the highest adsorption capacity for Hg^2+^ (Qmax, 11.722 mg·g^−1^), which was 1.8 times that of the B (6.541 mg·g^−1^, Table 3). One study showed that the adsorption capacity of soybean straw based biochar for mercury in aqueous solution was 0.674 mg·g^−1^ [25], and another study showed that the adsorption capacity of granular bentonite for Hg^2+^ was 1.7 mg·g^−1^ [26], which were only equivalent to 5.75% and 14.5% of the CB adsorption capacity, respectively. Obviously, the data from this showed that the CB produced by the combination of biochar and bentonite greatly improved the adsorption of Hg^2+^. Another study showed that the adsorption capacity of a graphene and biochar composite for mercury in aqueous solution reached 25.3 mg·g^−1^ [27], although the adsorption capacity of the CB for Hg was only 46.33% of that of the CB. In addition, compared with graphene sheet, the CB produced using the local millet straw and bentonite composite was much cheaper and easier to obtain in large quantities. Therefore, CB may be an excellent candidate material for effective and low cost support of carbon composites in local environmental material applications.

Compared with the B, the adsorption capacity of the MB to Hg^2+^ (Qmax, 9.152 mg·g^−1^) was also relatively high, indicating that potassium hydroxide and hydrochloric acid etching may have been key factors for an increase in the CB adsorption capacity after composite modification. Some studies have shown that hydrochloric acid and sodium hydroxide etching can increase the specific surface area and the number of micropores in the biochar adsorbent, thus improving the adsorption effect [18,19,28]. It has also been shown that the high-energy adsorption sites of metal ions are primarily located on the edges, defects, and vacancies of the oxide interlayers [29,30,31]. However, as reflected by the SEM images, after the bentonite and biochar were compounded, ribbed curly aggregates formed on the CB surface (Figure 1d). After the CB fully adsorbed Hg^2+^, the nanoscale linear chain became clearer and more obvious, and the uniform distribution was due to Hg^2+^ adsorbed and fixed at both ends of the CB interlayer, thus increasing the chain length and enhancing the exposure of the linear chain (Figure 1e).Therefore, it is speculated that many adsorption sites on the edge and interlayer of the CB were composed of polar adsorption sites of the original biochar and nonpolar adsorption sites of the bentonite. Because there are many types of adsorption sites for Hg^2+^, the adsorption capacity of the CB for Hg^2+^ was higher. The MB had a stronger binding affinity than the B (as evidenced by the values of b and the Kf value, Table 3). The experimental results showed that the adsorption efficiency of the MB for Hg^2+^ was greater than that of the B, although it has been reported that bentonite can adsorb heavy metal ions in aqueous solution in large quantities [32]. However, in this experiment, its adsorption capacity (Qmax, 2.013 mg·g^−1^) was much smaller than that of the B, MB, and the CB.

#### 3.2.3. Thermodynamic Adsorption of Hg^2+^ by the CB

The values of the thermodynamic parameters of the CB are shown in Table 4. ΔH > 0 indicates that the reaction was an endothermic process, that is, the adsorption capacity increased gradually with an increase in the temperature (Figure 3c). The linear regression between ln(k) and 1/T was not completely linear (Figure 3d), which indicated that adsorption was not only chemical adsorption, but may have been accompanied by a partial physical adsorption. The Gibbs free energy (kJ·mol^−1^) values calculated at three temperatures (15 °C, 25 °C, and 35 °C) were all negative values, and the absolute values became larger and larger with an increase in the temperature. This result further verified that the adsorption process of Hg^2+^ by CB was an endothermic reaction and an increase in the temperature was beneficial for adsorption. The entropy of the Hg^2+^ adsorption process of the composite material became positive, which indicated that the adsorption process was a thermodynamic process of increasing entropy and enthalpy. Therefore, according to the thermodynamic principle, the adsorption process was a spontaneous process at high temperature, that is, increasing temperatures were beneficial to the adsorption process.

#### 3.2.4. Effect of Initial pH on Adsorption of Hg^2+^ by CB

pH plays an important role in adsorption, which can change the adsorption capacity of adsorbents by affecting the amount of surface charges of the adsorbent and the ionization degree of Hg^2+^ [33,34]. In the range of pH value of 2–8, the main purpose is to avoid Hg^2+^ easily combining with OH^−^ in aqueous solution to precipitate when the pH of the solution is high [35]. As can be seen from Figure 3e, when the pH value increases from 2 to 7, the adsorption amounts of B, MB and CB all increase with the increase of pH value. At pH = 7, the adsorption capacity of CB and MB reached the maximum, 0.974 (97.4%) and 0.926 (92.6%) mg·g^−1^, respectively, which was much higher than that of B and BE. When pH < 4, the adsorption capacity of all adsorbents is low, which mainly because of the competitive adsorption between high concentration of H^+^ and Hg^2+^. On the other hand, the high concentration of H^+^ inhibits the adsorption of OH^−^ and Cl^−^ by the adsorbent, thus inhibiting the complexation with Hg^2+^. With the increase of pH value, more negatively charged ligands such as carboxyl functional groups are exposed, and positively charged Hg^2+^ is adsorbed on the remaining surface, which is beneficial to adsorption. Interestingly, when pH is equal to 8, the adsorption capacity of Hg^2+^ by CB decreases slightly. It is worth noting that BE was much lower than other adsorbents such as CB in adsorption experiments. The possible reason is that bentonite has fine particles and micropores are easily blocked in the process of Hg adsorption [36,37,38], while CB has the common characteristics of biochar and bentonite, forming a bimodal adsorption system with mesopores leading to micropores [11], so it has higher adsorption capacity for Hg^2+^, and its adsorption mechanism for Hg^2+^ needs further experimental study.

### 3.3. Adsorption Mechanism of the CB on Hg^2+^

#### 3.3.1. FTIR

**Figure 4:**
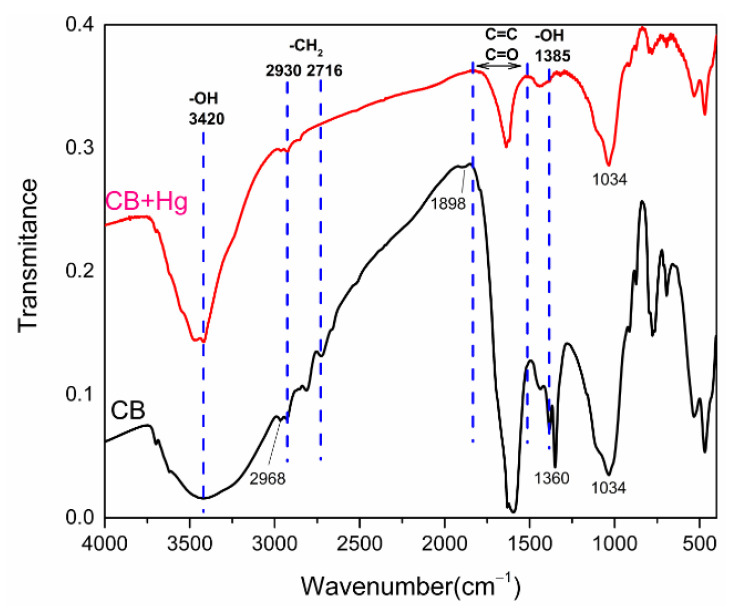
The FTIR spectra of CB before and after Hg^2+^ adsorption.

To clarify the adsorption mechanism, Fourier transform infrared spectroscopy was performed to compare the difference between the adsorption and non-adsorption of Hg^2+^ on the CB (Figure 4). The O–H bending vibration, which was within the peak at 1385 cm^−1^ [39], was greatly weakened and nearly disappeared after the adsorption of Hg^2+^ by the CB. This indicates that the adsorption of Hg^2+^ with the hydroxyl group of CB consumed the hydroxyl group of the CB. The wide spectral band near 3420 cm^−1^ was related to the v (OH) vibration group in the hydroxyl group [40,41]. The wide peak pattern at 3420 cm^−1^ in the CB indicated that the intermolecular –OH associated with the hydrogen bond before adsorption was the primary existing form of –OH in the CB. After the adsorption of Hg^2+^, the narrowing of the peak pattern indicated that adsorption of Hg^2+^ consumed part of the intermolecular –OH in the CB, and the remaining –OH consisted primarily of intramolecular association. Therefore, it can be concluded that intermolecular –OH played an important role in the adsorption of Hg^2+^ in the CB.

The peaks at 2930, 2803, and 2716 cm^−1^ corresponded to the deformation vibration of the asymmetric stretching peak of –CH_2_ [39]. The peaks at 2930, 2803, and 2716 cm corresponded to the deformation vibration of the CH_2_ asymmetric stretching peak [39]. Peaks at 1928–1550 cm^−1^ belonged to the adsorption area of C=O/C=C, where the peak value was greatly reduced, which indicated that the CB consumed a lot of C=O/C=C when adsorbing Hg^2+^. Hence, C=O/C=C played an important role in the adsorption process of Hg^2+^ by the CB. Previous studies have shown that heavy metal ions adsorbed on biochar adsorbents can be combined with heavy metals through the π bond structure of the double bonds [41]. Therefore, it is speculated that the π bond plays an important role in the adsorption of Hg^2+^ by the CB.

#### 3.3.2. XPS

Figure 2c,d are the X-ray photoelectron spectroscopy of the CB before and after Hg^2+^ adsorption. After Hg^2+^ adsorption by the CB, it was observed that the intensity of Hg4f increased obviously from the wide scanning spectrum (Figure 2e). After Hg^2+^ was adsorbed by the CB, the spectral intensity of O1s decreased (Figure 2e). Figure 2f shows the composition and morphology of the Hg element on the surface of the CB after adsorption. The binding energies of the XPS typical absorption peaks of Hg^2+^ are 102.2 eV and 104.2 eV [39,42]. The molar ratios of these two mercury compounds on the CB surface were 71.23% and 28.77%. Although the current references only indicated that the binding energies of these two kinds of mercury are different, Hg was fractionated into two kinds from a single Hg^2+^, suggesting that the existence of these two different peaks may have been due to the different adsorption of Hg by the oxygen-containing groups of the CB. This further indicates that the oxygen-containing functional groups on the CB surface had two different binding effects on Hg^2+^.

Figure 2c,d show the changes in the oxygen-containing functional groups before and after adsorption, and the molar ratio of Si–O barely changed, which indicates that the group did not play a role in the adsorption of Hg^2+^. The molar ratio of C–O increased (30.61–45.71%), while the molar ratio of C=O decreased (40.24–26.03%), indicating that the physical adsorption of Hg^2+^ by CB also occurred during the complexation reaction. C=O combines with Hg to generate C–O–Hg. Hg reacts with oxygen in the form of complexation, forming a single-tooth or double-tooth complex (Hg–O– or Hg=O), and at the same time, C=O is converted into C–O. The decrease in the molar ratio of –OH (11.92–8.41%) indicates that when the oxygen in the intermolecular –OH in the CB was combined with mercury, hydrogen and hydroxyl were further combined to form water molecules, thus generating O–Hg and consuming a proportion of the hydroxyl. The molar ratio of Hg–O was 2.43%, which also verifies the above description. Therefore, combined with the FTIR results, the adsorption of Hg^2+^ by CB included two types: one was hydroxyl-dominated adsorption and complexation, and the other was C=O dominated internal tooth complexation. In the carbon map, the molar ratio of C=O decreased, and the molar ratio of C–O increased after the adsorption of Hg^2+^ by the CB. This suggested that the C=O reacted with mercury to form C–O–Hg. The appearance of C–O–O and the increase in the C–Cl molar ratio also verifies the above conclusions (Figure 2g,h).

#### 3.3.3. TGA

**Figure 5:**
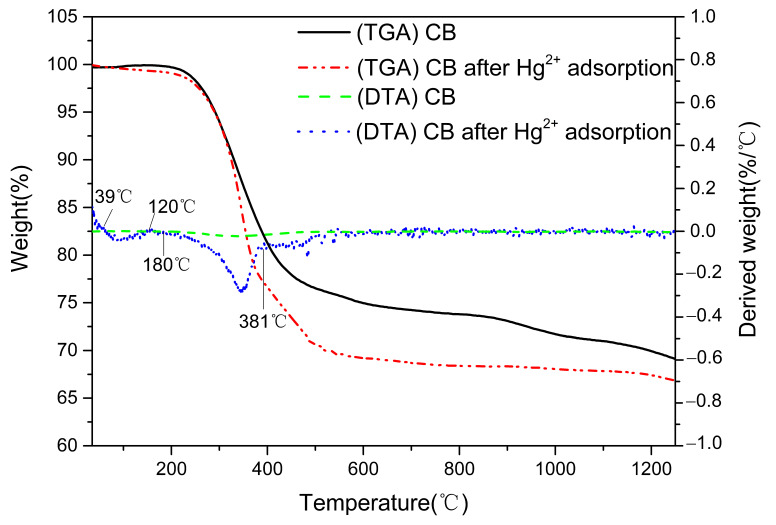
The TGA and DTG curves of the CB before and after Hg^2+^ adsorption.

A thermogravimetric analysis test was conducted to compare the difference between adsorption and non-adsorption of Hg^2+^ on the CB (Figure 5), and the results are shown in Figure 5. With the adsorbed Hg^2+^, CB showed a new peak on the DTG (differential thermal gravity) curve between 180 °C and 381 °C (Figure 5) that was attributed to thermal decomposition of HgO [43]. Hg_2_O is formed at the interface of two solid phases (HgO/Hg_2_O), and the free atoms of mercury are oxidized by oxygen atoms at low temperatures (<230 °C). The formation of HgO is related to the formation of the reaction interface, which separates two solid phases (HgO/Hg_2_O) and transfers the generation energy of the partially separated Hg_2_O to the reactants, thus improving the decomposition of HgO [44]. Therefore, the weight loss between 39 and 120 °C should be considered as the thermal decomposition of Hg_2_O. The above contents further confirm that the adsorption mechanism of Hg^2+^ by the CB played an important role due to the oxygen of the CB.

HgO (s)→Hg (g) + O

Hg_2_O (s)→HgO (s) + O

**Figure 6:**
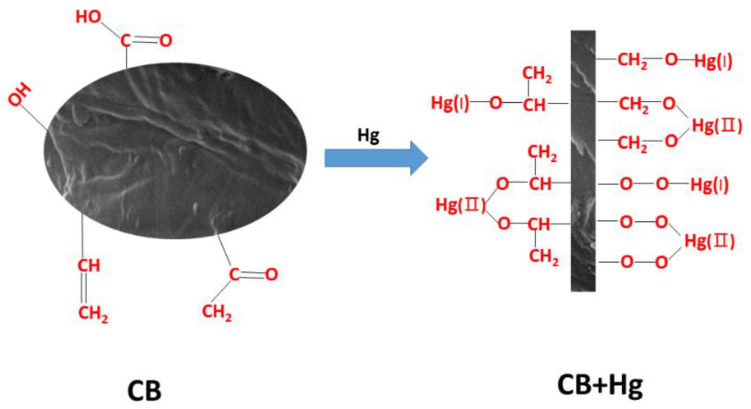
Schematic illustration of the possible Hg adsorption mechanism of the CB.

According to the above, it can be concluded that the Hg^2+^ in the solution was adsorbed by the –OH or the π bond and then combined with the –OH, C=O, and COO^−1^ of CB. It was then transformed into the Hg–O/Hg–O–Hg monodentate or bidentate compound. The possible mercury adsorption mechanism of the CB is shown in Figure 6.

## 4. Conclusions

In the nitrogen atmosphere, a new adsorbent (a biochar/bentonite composite called CB) was synthesized by compounding and modifying millet straw biochar and bentonite using solution intercalation. The CB showed enhanced physical and chemical properties, such as a larger surface area, pore diameter and pore volume, a more neutral carbon and oxygen content, and an excellent adsorption capacity for mercury. Its synthesis was relatively simple and easy, and it possessed a good adsorption performance. These results indicated that it has the potential to adsorb and remove inorganic pollutants in many environmental applications. This method provides a theoretical basis for the removal of heavy metals in polluted water environment.

## Figures and Tables

**Table 1:** Physiochemical properties of the four kinds of adsorbent selected.

Adsorbent	C (%)	H (%)	S (%)	N (%)	O (%)	Ash Content (%)	S_BET_ (m^2^/g)	Pore Volume (cm^3^/g)	Pore Size (nm)
B	84.9	4.77	0.45	2.66	17.6	7.8	86.7	0.032	64.1
BE	1.4	0.52	0.21	1.49	41.3	90.5	52.5	0.106	85.2
MB	55.1	3.85	0.27	2.05	21.2	14.2	869.2	0.712	62.5
CB	17.2	1.55	0.35	0.92	28.5	25.4	945.33	0.788	44.5

**Table 2:** The kinetic model parameters of Hg^2+^ on the adsorbent.

Adsorbent	Pseudo First-Order Dynamic Mode			Pseudo Second-Order Dynamic Mode		
	qe (mg·g^−1^)	k1 (/min)	R^2^	qe (mg·g^−1^)	K2 (/min)	R^2^
B	0.551	0.251	0.964	0.603	0.582	0.981
BE	0.237	1.393	0.859	0.245	11.756	0.994
MB	0.872	0.413	0.959	0.933	0.682	0.936
CB	0.989	0.72	0.916	1.044	1.16	0.988

**Table 3:** The regression parameters of the isothermal adsorption model.

Adsorbent	Langmuir			Freundlich		
	Qmax	b	R^2^	Kf	n	R^2^
B	6.541	0.328	0.995	1.725	2.204	0.987
BE	2.013	0.125	0.984	0.293	1.803	0.995
MB	9.152	0.608	0.992	3.223	2.505	0.949
CB	11.722	0.749	0.991	4.505	2.482	0.981

**Table 4:** The linear regression of the adsorption thermodynamic model of Hg^2+^ for the CB.

T (K)	ΔG	ΔH	ΔS
288.15	−3.52	-	-
298.15	−7.14	11.75	29.5
308.15	−9.39	-	-

## Data Availability

The data presented in this study are available on request from the corresponding author.

## Supplementary Materials

The following are available online at https://www.mdpi.com/1996-1944/14/5/1117/s1, Figure S1: The Single-factor Hg adsorption experiments with 5 factors and 3 levels studied. Table S1: Different level values of CB preparation conditions. Table S2: Results of orthogonal test.
