# Supplementary material for: Preparation of a Novel Millet Straw Biochar-Bentonite Composite and Its Adsorption Property of Hg2+ in Aqueous Solution
Source: Materials (Basel). 2021 Feb 27;14(5):1117. doi: 10.3390/ma14051117 (PMC7957562; doi:10.3390/ma14051117)
Supplement: Supplementary file 1 [file materials-14-01117-s001.pdf]

materials

MDPI

Supplementary Materials

Preparation of a Novel Millet Straw Biochar-Bentonite Composite and its Adsorption Property of Hg^{2+} in Aqueous Solution

Text. Optimization of preparation conditions of CB

Prepare 5 mg L^{-1} Hg solution (calculated as Hg), add 1 g L^{-1} of different samples, shake for 30 min, centrifuge, take 50–100 μL supernatant, measure Hg content with DMA-80 and calculate Hg adsorption value of CB. The experimental data were statistically analyzed by Excel 2017 and IBM SPSS22.0 (SPSS22.0, IBM, New York, NY, USA). The new complex polarization method was used to test the significant difference, and origin 8.2 (OriginLab, Northampton, UK) was used for mapping.

Particle size of straw, mass concentration of KOH solution, mixing ratio of straw biomass and bentonite, reaction temperature and carbonization temperature of straw powder and bentonite were investigated. The values of different factors and levels are listed in table S1.

There are five factors studied, and each factor has three levels, so L_{27} (3^{13}) orthogonal test table is selected. The design table and results of orthogonal test are listed in Table S1.

The results of orthogonal test are listed in Table S2. From Table S2, it can be seen that various factors have different influences on CB's ability to absorb Hg^{2+}. According to the analysis method of orthogonal test, the maximum average value among different levels represents the best level of this factor. Therefore, the best conditions of five factors are as follows: the grain size of millet straw is 0.074 mm; Potassium hydroxide concentration is 3 mol·L^{-1}; Straw: bentonite (w: w) = 2: 1; The reaction temperature (°C) is 70; The carbonization temperature (°C) is 300 °C. In this study, the maximum Hg adsorption capacity is the reference standard for good CB adsorption performance. Compared with the repeated experimental results of Ex12 (Hg adsorption value is 3457.402 mg·kg^{-1}) and the above-mentioned optimal conditions (Hg adsorption value is 3970.561 mg·kg^{-1}), it is concluded that the best condition $A_1B_2C_3D_3E_2$ can produce CB with larger adsorption capacity of Hg^{2+}. Then, according to the experimental conditions of $A_1B_2C_3D_3E_2$, further single factor experiments were carried out.

According to the maximum adsorption value of Hg in different single factor experiments, the absorption capacity can be judged. Figure S1 shows that the best condition of single factor experiment is $A_2B_2C_3D_2E_3$. However, compared with the repeated experimental results of $A_1B_2C_3D_3E_2$ (Hg adsorption value is 3435.822 mg·kg^{-1}) and the optimal conditions of the single factor experiment (Hg adsorption value is 3016.557 mg kg^{-1}), it is confirmed that the experimental conditions of $A_1B_2C_3D_3E_2$ will produce composite materials with larger Hg^{2+} adsorption capacity. Therefore, the optimum technological conditions are as follows: straw powder size (0.074 mm), potassium hydroxide concentration (3mol·L^{-1}), straw: bentonite = 2:1 (w/w), reaction temperature (70 °C) and carbonization temperature (300 °C).

Table S1. Different level values of preparation conditions.

Level	Straw Particle Size (mm)	KOH Concentration (mol·L^{-1})	Straw/Bentonite (w/w)	Reaction Temperature (°C)	Carbonization Temperature (°C)
1	0.074	1	1:2	30	200
2	0.45	3	1:1	50	300
3	1	5	2:1	70	400

Table S2. Results of orthogonal test.

Treatment	A	B	C	D	E	F
Ex1	0.074	1	1:2	30	200	275.65
Ex2	0.074	1	1:2	30	300	2062.87
Ex3	0.074	1	1:2	30	400	487.87

Ex4	0.074	3	1:1	50	200	3146.48
Ex5	0.074	3	1:1	50	300	3423.32
Ex6	0.074	3	1:1	50	400	3300.82
Ex7	0.074	5	2:1	70	200	3240.85
Ex8	0.074	5	2:1	70	300	2415.14
Ex9	0.074	5	2:1	70	400	3138.54
Ex10	0.45	1	1:1	70	200	1290.26
Ex11	0.45	1	1:1	70	300	2564.46
Ex12	0.45	1	1:1	70	400	3851.34
Ex13	0.45	3	2:1	30	200	2655.37
Ex14	0.45	3	2:1	30	300	2914.68
Ex15	0.45	3	2:1	30	400	1273.04
Ex16	0.45	5	1:2	30	200	809.39
Ex17	0.45	5	1:2	50	300	935.54
Ex18	0.45	5	1:2	50	400	460.35
Ex19	1	1	2:1	50	200	2238.30
Ex20	1	1	2:1	50	300	2276.10
Ex21	1	1	2:1	50	400	2148.10
Ex22	1	3	1:2	70	200	2752.35
Ex23	1	3	1:2	70	300	2399.04
Ex24	1	3	1:2	70	400	1141.99
Ex25	1	5	1:1	30	200	2344.39
Ex26	1	5	1:1	30	300	477.69
Ex27	1	5	1:1	30	400	1147.23
K1	2387.949	1910.550	2393.999	1515.421	2083.671	-
K2	1861.603	2556.343	1258.339	2082.044	2163.204	-
K3	1880.577	1663.236	2477.791	2532.663	1883.253	-
Range	526.346	893.108	1219.452	1017.242	279.951	-

Note: F: Hg adsorption value (mg·kg^{-1}). K: Average value of Hg adsorption value.

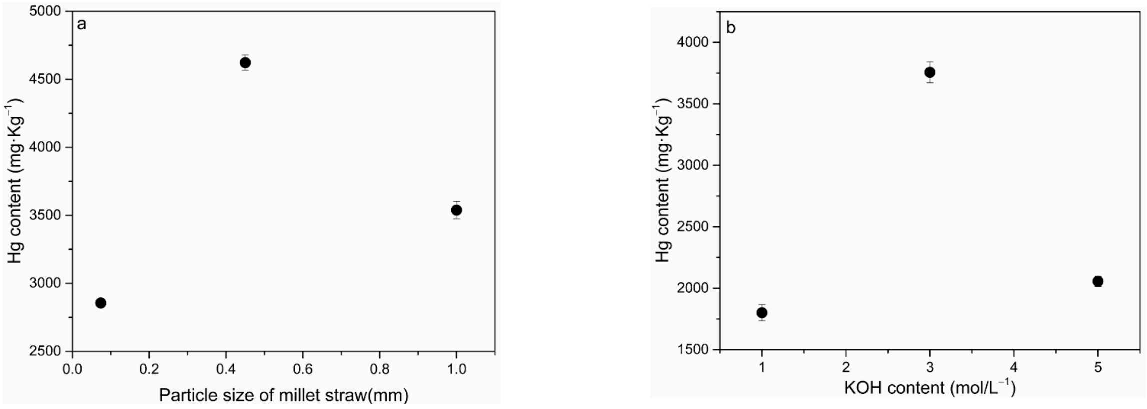

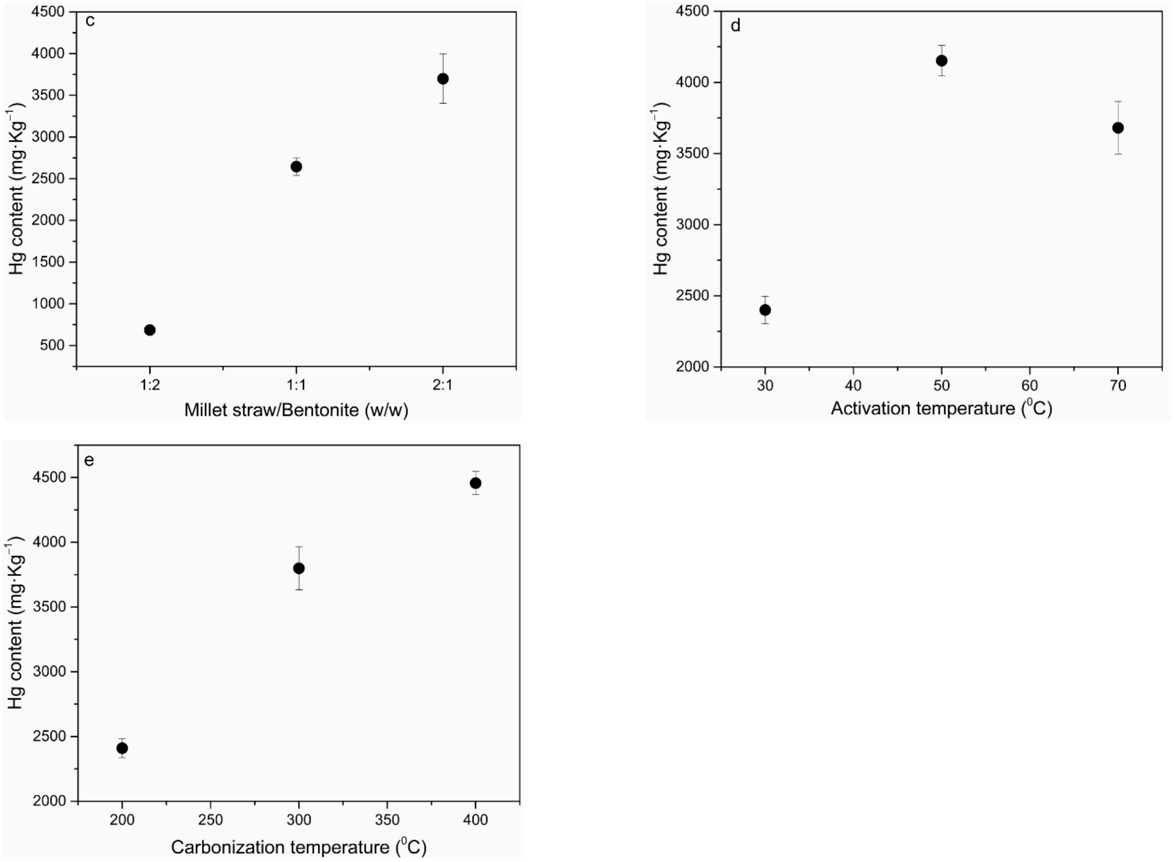

Figure S1. The Single-factor Hg adsorption experiments with 5 factors and 3 levels studied. (**a**) Single-factor experiments of particle of millet straw, (**b**) Single-factor experiments of KOH content, (**c**) Single-factor experiments of millet straw/bentonite, (**d**) Single-factor experiments of activation temperature, (**e**) Single-factor experiments of carbonization temperature.
